# Green Synthesis, Molecular Characterization and Associative Behavior of Some Gemini Surfactants without a Spacer Group

**DOI:** 10.3390/ma6041506

**Published:** 2013-04-12

**Authors:** Carla Villa, Sara Baldassari, Delia F. Chillura Martino, Alberto Spinella, Eugenio Caponetti

**Affiliations:** 1 DIFAR—Department of Pharmacy, University of Genoa, Viale Cembrano 4, 16147 Genoa, Italy; E-Mail: s.baldassari@unige.it; 2 STEBICEF—Department of Biologic, Chemical and Pharmaceutical Science and Technology, University of Palermo, Viale delle Scienze, P.co d’Orleans II, Pad. 17-I90128 Palermo, Italy; E-Mails: delia.chilluramartino@unipa.it (M.D.); eugenio.caponetti@unipa.it (E.C.); 3 Centro Grandi Apparecchiature-UniNetLab, University of Palermo, Via F. Marini, 14-I90128 Palermo, Italy; E-Mail: alberto.spinella@unipa.it

**Keywords:** gemini surfactants, green synthesis, microwave, cosmetic detergents, critical micelle concentration, PFG-NMR, diffusion coefficient

## Abstract

A series of new gemini surfactants without a spacer group, disodium 2,3-dialkyl-1,2,3,4-butanetetracarboxylates, were synthesized in a green chemistry context minimizing the use of organic solvents and applying microwaves (MW) when activation energy was required. Once the desired architecture was confirmed by means of the nuclear magnetic resonance technique (^1^H-NMR, ^1^H-^1^H COSY) for all the studied surfactants, the critical micellization concentration was determined by conductance measurements. The diffusion coefficient of micelles formed by the four compounds was characterized using pulsed field gradient (PFG)-NMR. Diffusion coefficients were found to be dependent on the concentration and on the number of carbon atoms in the alkyl chain. The absence of the spacer group, peculiar to this new series of gemini surfactants, may confer relatively low flexibility to the molecules, with potential implications on the interfacial properties, namely on micellization. These gemini surfactants might have interesting applications in the preparation of composite materials, in nanotechnology, in gene transfection and mainly, due to the low CMCs, as new interesting ingredients of cosmetics and toiletries.

## 1. Introduction

In recent years, research has been widely carried out on gemini surfactants, due to their advantages over monomeric surfactants with respect to different applications. The chemical structure of geminis, consisting of two amphiphilic moieties and two polar head groups generally connected by a spacer group, makes them a class of compounds with its own distinct behavior. The critical micelle concentration (CMC) of gemini surfactants is typically one or two orders of magnitude lower than the one of the corresponding monomeric surfactant [1,2]; this feature may be related to the lower environmental impact of the molecule, because a much smaller quantity will be required to perform the same function when compared to conventional surfactants. Gemini surfactants are of interest for cosmetics and toiletries, in particular shampoos and personal care products, because of their mildness, soft feeling and low skin irritation [3]. These surfactants often possess excellent wetting, dispersing, emulsifying and cleaning properties. Many patents on cosmetic compositions have been issued in the latest years containing gemini surfactants, either non-ionic or ionic; in particular, the Japanese market has been expressing deep interest in this kind of ingredient. Recently, gemini surfactants have shown outstanding properties, as they apparently self-organize in water, forming a nano-order vesicle structure, which might be important for enhanced humectant function and increasing the skin permeability of effective components [4].

Other applications are in the preparation of composite materials, in nanotechnology, in cellular transfection, *etc*.

The CMC value of geminis often varies not linearly with the number of carbon atoms in the surfactant alkyl chain, because of a pre-micellar aggregation at concentrations lower than CMC. This effect becomes significant for a large number of carbon atoms in the alkyl chains [5,6]. The CMC depends mainly on the length and nature of the spacer group [7], which seems to be the most important parameter in determining the properties of gemini surfactant micellar solutions and of the microstructure of micelles [8]. Many reviews concerning the synthesis and the structure of gemini surfactants have been published. Novel molecular architectures differing with regard to the polar head groups, the hydrocarbon chains and the type and size of the spacers have been proposed [9,10].

Taking into account these previous statements, our attention has focused on a specific anionic gemini surfactant lacking the spacer group, namely, disodium 2,3-didodecyl-1,2,3,4-butanetetracarboxylate [11] (**4c**), whose CMC and phase behavior have already been reported in the literature [12,13]. 

**Figure 1:**
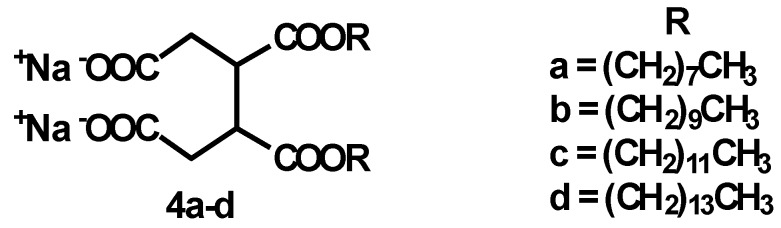
Disodium 2,3-dialkyl-1,2,3,4-butanetetracarboxylates **4a–d**.

Hence, a series of disodium 2,3-dialkyl-1,2,3,4-butanetetracarboxylates, with alkyl chain lengths from 8 to 14 C (compounds **4a–d**, Figure 1), have been synthesized using a microwave-assisted eco-friendly procedure, and several properties of the molecules have been analyzed with the aim of evaluating the influence of the alkyl chain on the CMC value and of investigating the structure of the aggregates these surfactants form in aqueous medium.

The conventional procedures reported in the literature for the synthesis of gemini surfactants [8,9], including the synthetic pathway for the above-mentioned compound, **4c** [10], require organic solvents, strong acidic reagents, long reaction times and difficult work-up. On the basis of these considerations, the synthesis of the studied compounds was carried out according to the Green Chemistry principles, minimizing the use of organic solvents and applying microwaves (MW) as an alternative energy source when activation energy was required.

The purity and the molecular structure of the synthesized compounds were established by IR, ^1^H-NMR and ^13^C-^1^H HSQC analyses. Once the desired architecture was confirmed, the critical micelle concentration was determined for all surfactants by conductance measurements with the aim of evaluating the influence of the alkyl chain length on the surfactant behavior.

The translational diffusion coefficient of the micelles formed by the four compounds (**4a–d**) in aqueous solutions as a function of the concentration was obtained by pulsed field gradient (PFG) NMR, which is a proven tool for the quantitative measurement of the diffusion coefficient. Its usage has increased considerably over the past decade with the introduction of reasonably linear, self-shielded gradient coils in high resolution NMR probes. This makes it relatively straightforward to obtain quantitative translational diffusion coefficients for a wide variety of diffusing objects, ranging from solvent molecules to large particles or aggregates.

The recorded data have been analyzed for a specific characterization of these new molecules with regard to the potential applications in various fields, especially the cosmetic one.

## 2. Results and Discussion

### 2.1. Green Synthesis of Disodium 2,3-Dialkyl-1,2,3,4-butanetetracarboxylates, **4a–d**

**Scheme 1:**
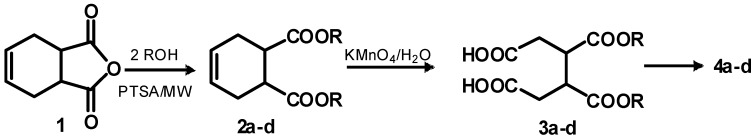
Synthetic steps for gemini surfactants **4a-d**.

The bis-anionic gemini surfactants, compounds **4a–d**, were obtained in good yields using an eco-friendly procedure applying the synthetic route, consisting of two steps, reported in Scheme 1.

The first step was carried out by a fast solvent-free procedure under microwave activation to obtain compounds **2a–d** (alkylcyclohex-4-ene-1,2-dicarboxylates). Stoichiometric amounts of *cis*-1,2,3,6-tetrahydrophtalic anhydride **1** and the opportune alcohol (**a**: 1-octanol; **b**: 1-decanol; **c**: 1-dodecanol; **d**: 1-tetradecanol) were mixed in the presence of *p*-Toluenesulfonic acid as the acidic catalyst, without any solvent, and irradiated for 15 minutes at 110 °C. Yields of purified intermediates range from 79% to 82%.

In order to evaluate the influence of microwave activation on reaction efficiency, compound **2c**, as an example, was also synthesized under conventional heating (Δ, thermostated oil bath) at the same conditions (amount of reagents, temperature, vessel, stirring and reaction time) as under microwave irradiation. The yield obtained by this synthetic pathway was appreciably lower (yield % MW/Δ = 82/62).

In line with the green chemistry targets, in the second step, water was selected as the solvent to promote the oxidative cleavage of the double bond of the diesters, **2a–d,** with KMnO_4_, to obtain the 2,3-dialkyl-1,2,3,4-butanetetracarboxylate acids, **3a–d.** The reaction mixture was maintained under stirring at room temperature for 16 h.

### 2.2. Characterization

#### 2.2.1. ^1^H-NMR and ^1^H-^1^H COSY

**Figure 2:**
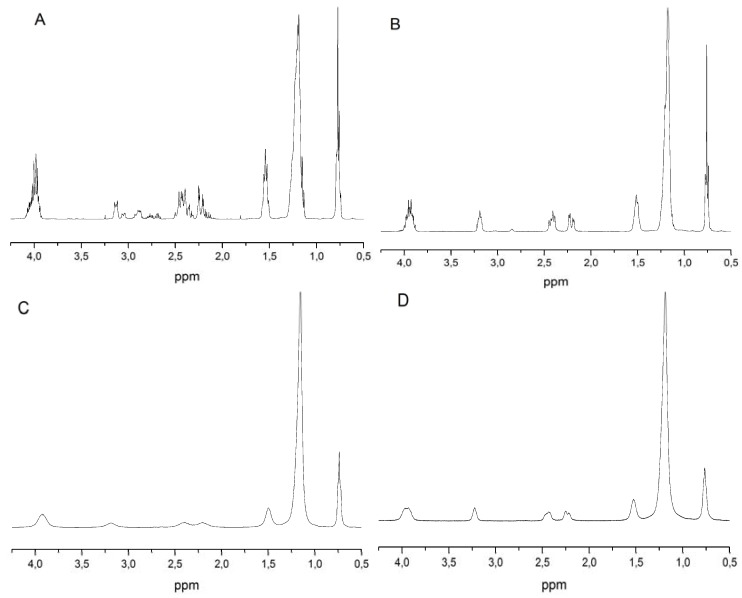
(**A**) ^1^H-NMR spectrum of **4a**; (**B**) ^1^H-NMR spectrum of **4b**; (**C**) ^1^H-NMR spectrum of **4c**; (**D**) ^1^H-NMR spectrum of **4d**.

The identity of compounds **4a–d** was confirmed through ^1^H nuclear magnetic resonance spectroscopy (^1^H-NMR). The ^1^H-NMR spectra of all compounds in deuterium dioxide are reported in Figure 2. 

All spectra display the same sequence of resonance signals. Protons in the aliphatic chain resonate below 2 ppm where three signals are present. The signals at 2.4 and 2.2 ppm are attributable to the protons of the CH_2_ groups bonded to the carboxylic groups. The signal at 3.2 ppm is attributable to the -CH- group. Finally, the protons of the CH_2_ group bonded to the oxygen atom resonate at 3.95 ppm. Integrals of the signals and their relative chemical shift values are reported in Table 1. The integral values are in agreement with the surfactant molecular structure. In addition, the absence of other peaks in the spectra of compounds **4a–d** confirms the purity of the final compounds.

**Table 1:** Chemical shift values, integral of the signals and assignment to molecular groups derived from ^1^H-NMR spectra of compounds **4a–d**.

Chemical shift (ppm)	Protons number from integral	Chemical group
0.7	3	CH_3_
1.18	10(**4a**), 14(**4b**), 18(**4c**), 22(**4d**)	(CH_2_)_5_ compound **4a** (CH_2_)_7_ compound **4b** (CH_2_)_9_ compound **4c** (CH_2_)_11_ compound **4d**
1.5	2	CH_2_
2.4–2.2	2	CH_2_-COO
3.2	1	CH-COOR
3.95	2	CH_2_-O

**Figure 3:**
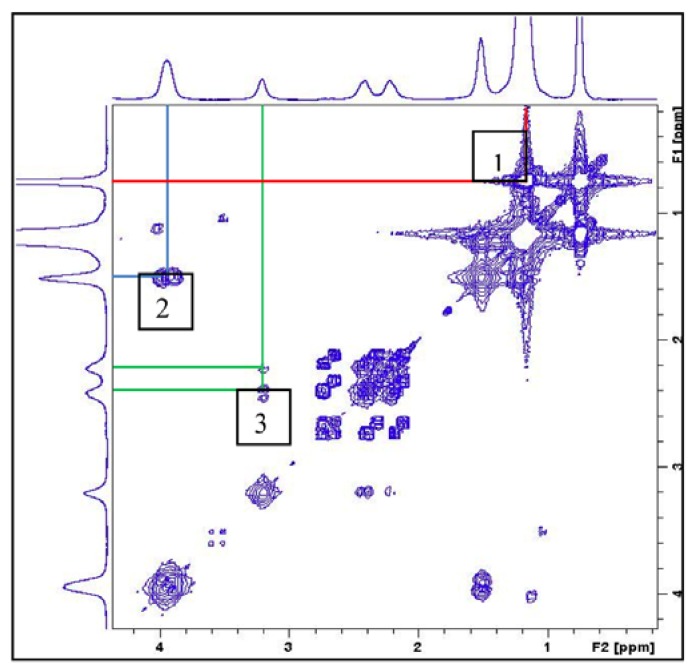
2D ^1^H-^1^H COSY of gemini surfactant **4c**.

As no multiplicity of the signals in the ^1^H-NMR spectra was observed, the 2D homonuclear correlation spectra (^1^H-^1^H COSY) were acquired in order to see the ^1^H-^1^H scalar couplings. As an example, the ^1^H-^1^H COSY correlation spectrum of compound **4c** is shown in Figure 3.

The lines whose crossing is labeled as 1 indicate the scalar coupling among protons of the terminal methyl group and the methylene groups of the alkyl chain, the ones whose crossing is labeled as 2 indicate the coupling of the methylene groups close to the ester oxygen and the ones whose crossing is labeled as 3, the coupling among the CH_2_ and CH groups in the polar head of the compound. The spectrum displays all the expected couplings, thus undoubtedly confirming the proposed structure. Equally, the 2D ^1^H-^1^H COSY correlation spectra of compounds **4a**, **4b** and **4c** confirm the proposed structures.

#### 2.2.2. Critical Micelle Concentration (CMC)

**Figure 4:**
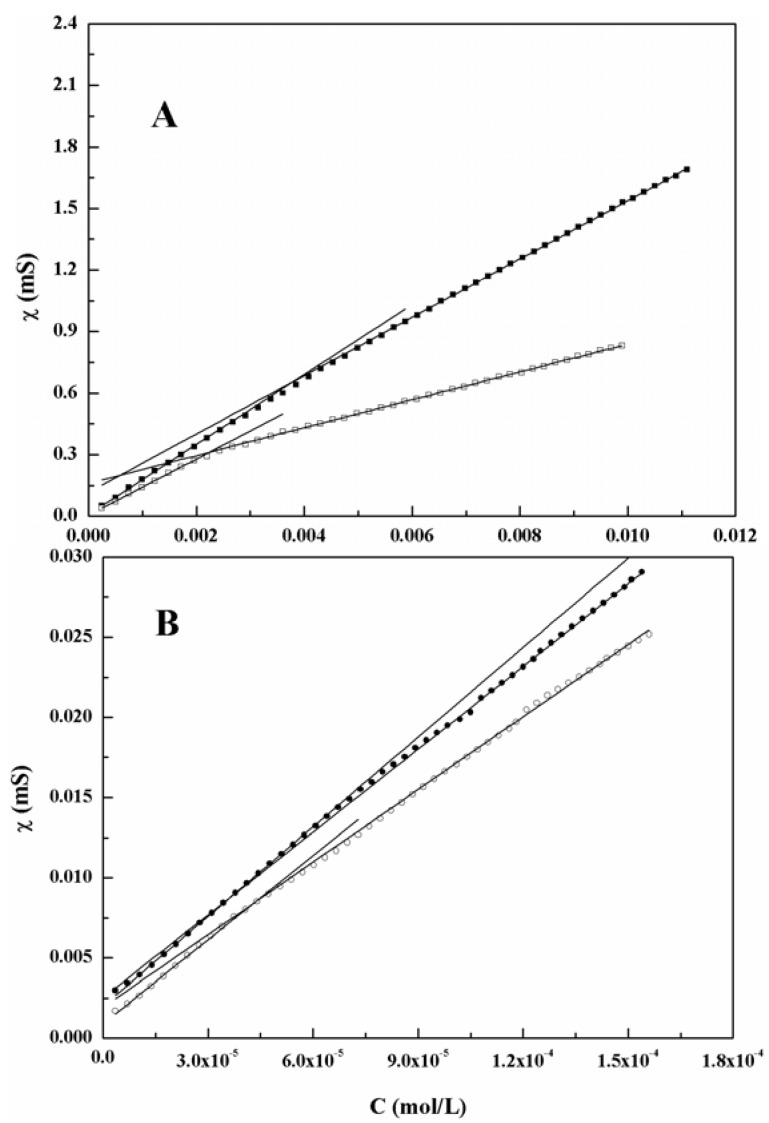
Conductivity values (χ) *vs.* molar concentration (C) for the gemini surfactants: (**A**) **4a** (solid symbol) and **4b** (open symbol); (**B**) **4c** (solid symbol) and **4d** (open symbol). The trend related to the compound **4c** solution has been shifted by 0.001 mS for reasons of clarity.

The CMCs of all synthesized surfactants were evaluated by investigating the variation of conductivity of the respective aqueous solution as a function of the surfactant concentration. The solutions were prepared by dissolving appropriate amounts of surfactant in deionized water (Ω = 18.1 MΩ). The conductivity values are displayed as a function of molar concentration in Figure 4. The experimental trend of each surfactant solution displays two linear regions having different slopes.

The intercept of the trends at low surfactant concentration tends to the value of 0.001 mS, which is the value measured for pure water. The presence of two sections with a different slope in the conductivity profile of each surfactant solution is attributed to the different conducting species. Actually, at low concentration, the anionic surfactant is molecularly dispersed and completely dissociated. This implies that the number of conducting species is elevated, and, due to their high ionic mobility, the increase of conductivity with the concentration is very rapid (as shown by the slope in the initial section of the profiles). At a higher concentration, several molecules aggregate to form micelles, and a certain number of counterions are associated to them within the Stern layer, thus screening the repulsive interactions among the charged head groups. Thus, the number of conducting species is lower than expected by considering the surfactant as completely molecularly dispersed. Moreover, these aggregates are bulkier than the free molecules and diffuse more slowly. Besides this, some surfactant molecules remain unassociated within micelles and contribute to the total conductivity even in this region. Therefore, the ionic conductivity still increases with the concentration, but the aggregation process justifies a lower slope. The CMC values were evaluated from the intersection of the two linear regions observed in the experimental conductivity profiles and are reported in Table 2. 

**Table 2:** Critical micelle concentration (CMC) determined by (**a**) conductivity measurements; and (**b**) by self-diffusion NMR for gemini surfactants **4a–d**. The error is on the last digit.

Surfactant	CMC (mol/L)
**4a**	5.1 × 10^−3 (a)^
**4b**	2.3 × 10^−3 (a)^ 1.53 × 10^−3 (b)^
**4c**	4.8 × 10^−5 (a)^
**4d**	7.8 × 10^−5 (a)^

**Figure 5:**
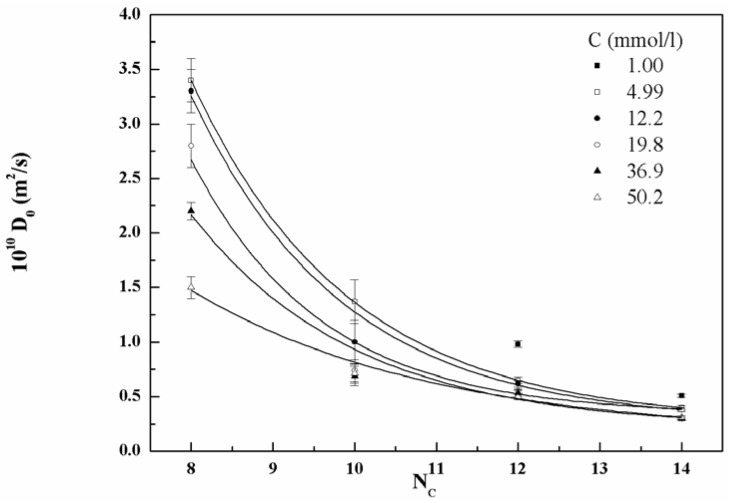
Diffusion coefficient (D_0_) *vs.* the number of carbon atoms (N_C_) in the alkyl chains of the gemini surfactants **4a–d** at various concentrations. Lines are an eye-guide only.

The CMC of compound **4c** results in fairly good agreement with data reported in the literature (8.9 × 10^−5^ by surface tension and 5.0 × 10^−5^ by the fluorescence-probe method) [12,13]. The CMC of these compounds decreases with the number of carbon atoms in the alkyl chains up to compound **4c**, then slightly increases for the surfactant, **4d**. The diffusion coefficients obtained by pulsed field gradient (PFG) NMR as functions of the concentration and the number of carbon atoms are shown in Figure 5.

**Figure 6:**
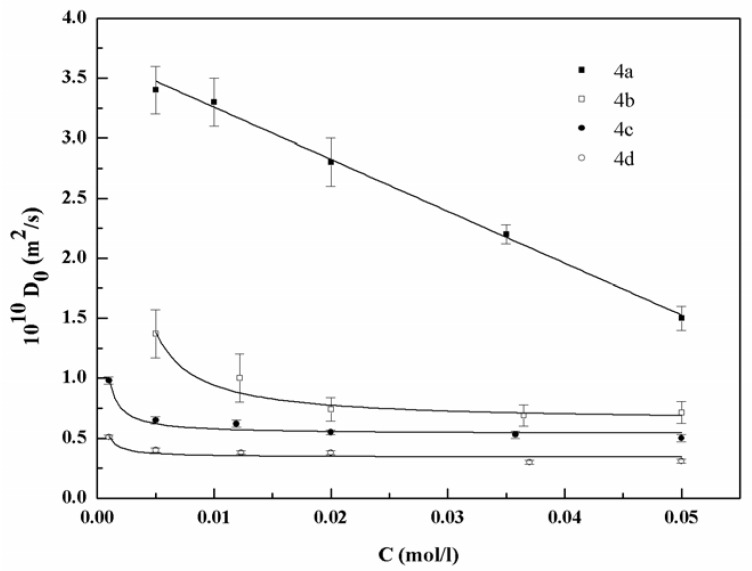
Diffusion coefficient (D_0_) *vs.* surfactant concentration (C) for surfactants **4a–d**. Lines represent the fits by the model described in the text.

The diffusion coefficient decreases upon increasing the number of carbons in the chains, indicating that the size of the aggregates increases. The observed trends for the four molecules are similar. The diffusion coefficient for surfactants **4a** and **4b** at 0.001 mol/L concentration (3.6 × 10^−10^ and 3 × 10^−10^ m^2^/s, respectively) was not shown on the same graph. This is because, being these concentrations lower than CMCs of both surfactants, the diffusion coefficient has to be ascribed to the presence of monomers in solution rather than to the micelles. In Figure 6, the values of D_0_ are also shown as a function of concentration for the four investigated surfactants in order to highlight the concentration dependence of the diffusion coefficient.

The obtained trends are similar for compounds **4b**, **4c** and **4d**, while the trend of compound **4a** is different. 

The translational diffusion coefficient depends on the size of the diffusing objects, *i.e.*, the surfactant in monomeric or associated form. In addition, the dependence of D_0_ on concentration and shape of the diffusing objects is to be considered. The effective translational mobility of a surfactant is considerably reduced when diffusing within a micelle, and the association process is reflected in a sensitive manner by the time-averaged self-diffusion coefficient. Applying the pseudo-phase separation model of micelle formation, at concentrations above the CMC, the monomer concentration is nearly constant, and the calculation of the experimental self-diffusion coefficients is based on the two-site model (micelles and free surfactant) [14]:

(1) $$D_{0}=p_{\text{mic}}D_{\text{mic}}+(1-p_{\text{mic}})\;D_{\text{free}}$$

where *D*_0_ has the same meaning as before; *D*_mic_ and *D*_free_ represent the translational diffusion coefficients of micellized and free surfactant, respectively, and *p*_mic_ is the fraction of micellized surfactant. To reproduce the relation between *D*_0_ and the total surfactant concentration, *C*_tot_, Equation (1) can be rewritten [15] using the Heaviside step function, H*(x)* = 0, when *x <* 0, and = 1, when *x >* 0:

(2) $$D_{0}=D_{\text{free}}+[1+(D_{\text{free}}-D_{\text{mic}})/D_{\text{free}}\cdot H(C_{\text{tot}}/\text{CMC}-1)\cdot [(\text{CMC}/C_{\text{tot}}-1)]$$

where CMC, *D*_free_ and *D*_mic_ are free-fitting parameters. The model here accounts for the size of aggregates, but does not account for their interaction and shape. The interactions among micelles derive from the electrical potential generated by the surface charge on the micelles and from the volume fraction of micellized surfactant. Detailed knowledge on the micelle structure is required to account for the above-listed terms. In the present case, it was assumed that the aggregates are weakly interacting and that their shape is nearly spherical. By using the above-described model, we were able to reproduce the trend for compounds **4b**, **4c** and **4d**. In particular, the fitting of Equation (2) to the trend of the **4b** surfactant allows us to evaluate its CMC. The value (1.53 × 10^−3 ^mol/L) is very close to the one determined by the conductivity method, thus confirming the quality of the model. Unfortunately, the evaluation of D_0_ in a range of concentration lower than the CMC was prevented by the long acquisition time and the high error on the single value; thus, the CMC values for compounds **4c** and **4d** were not determined. Consequently, in the fitting procedure, the CMC values were fixed to those determined by conductivity. The model above does not hold for surfactant **4a**. This implies that the pseudo-phase transition model does not hold for this surfactant. In fact, being the alkyl chain of compound **4a** short, the force balance between attractive and repulsive interactions can be able to maintain stably in solution aggregates of several aggregation numbers. This is in agreement with the mass-action model that considers the associative process as a combination of various equilibriums among two, three or more molecules with the consequence of generating aggregates of various aggregation numbers.

**Figure 7:**
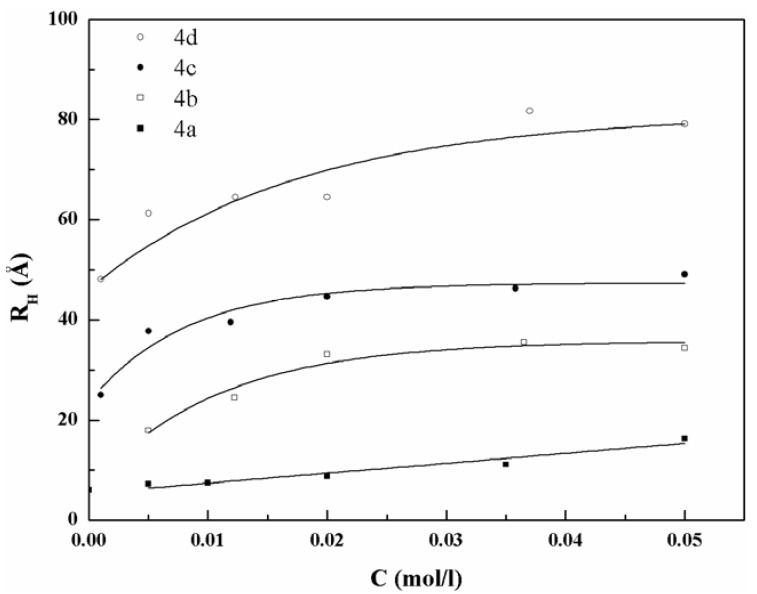
Hydrodynamic radius (R_H_) *vs.* concentration for the surfactants **4a–d**.

It is known that the *D_0_* of micelles diffusing in aqueous medium are related to their hydrodynamic radius, *R*_H_, evaluated by means of the Stokes-Einstein relation:

(3) $$D_{0}=kT/(6\text{πη}R_{\text{H}})$$

where *k* is the Boltzmann constant; *T* the temperature on absolute scale; and η the viscosity of the medium; *R*_H_ values computed by means of Equation (3) are shown in Figure 7. This simple model does not account for interaction among micelles and for their anisotropy. Other techniques, such as Small Angle Neutron Scattering, have to be used in order to attain information on the interactions among micelles, on their shape and on their internal structure.

However, the results indicate that the micelle sizes depend on the number of carbon atoms in the alkyl chains, and that the growth rate with the concentration is higher for longer compounds. The size growths with the concentration for compounds **4b**–**d** are similar to those observed for aqueous solutions of a large variety of monomeric surfactants [16,17,18,19,20]. 

## 3. Experimental Section 

### 3.1. Materials 

Diethyl ether was supplied by Merck (Darmstadt, Germany); Florisil 100–200 mesh, *p*-toluenesulfonic acid, H_2_SO_4_, KMnO_4, _*cis*-1,2,3,6-tetrahydrophtalic anhydride, 1-octanol, 1-decanol, 1-dodecanol and 1-tetradecanol were supplied by Sigma-Aldrich (Milan, Italy).

The microwave irradiation was carried out in a monomode reactor (Synthewave^TM^ S402 Prolabo), specially designed for organic syntheses. This apparatus allows an accurate control of the temperature by power modulation, temperature measurement by IR detection and mechanical stirring. 

IR spectra were registered using a Perkin-Elmer 398 spectrophotometer.

^1^H-NMR and ^1^H-^1^H COSY spectra were recorded on a Bruker II 400 spectrometer operating at 400.15 MHz for the ^1^H nucleus. 

The CMC of each compound was determined using a Conductimeter AMEL Mod. 160.

Diffusion ordered spectroscopy (DOSY) was detected with the above-mentioned NMR spectrometer, equipped with a field gradient probe unit. 

### 3.2. General Synthetic MW Procedures 

A simple mixture of reagents (10 mmol of the anhydride and 20 mmol of the appropriate alcohol) in the presence of *p*-toluenesulfonic acid (PTSA—20% by weight of the anhydride) as the catalyst was used. The mixture was introduced in a scientific monomode MW reactor and irradiated under mechanical stirring for 15 min at 110 °C as the final temperature. 

After cooling at room temperature, 20 mL of diethyl ether were added. The solution was washed with a NaHCO_3_ diluted solution and with deionized water, dried over MgSO_4_ and filtered. The solvent was evaporated under reduced pressure; the crude liquid products thus obtained (**2a–c**) were purified by elution (with diethyl ether) on a basic alumina column, while the solid compound, **2d** (m.p. 33–35 °C), was purified by recrystallization from 80 vol. % ethanol. Yields ranged from 79 to 82%.

The oxidative cleavage of the double bond of the diesters **2a–d**, to obtain 2,3-dialkyl-1,2,3,4-butanetetracarboxylic acids **3a–d**, was carried out using a KMnO_4_ aqueous solution (prepared by dissolving 4.74 g, 30 mmol, in 60 mL of H_2_O, without organic solvent or catalyst) added to the opportune diester (10 mmol, molar ratio diester/KMnO_4_ = 1:3) under high stirring in an ice bath. The reaction mixture was then kept under stirring at room temperature for 16 h; in the end, a saturated solution of Na_2_SO_3_ and a 6 M HCl solution were added, obtaining the decoloration of the mixture and the precipitation of the solid acid compound. The crude products were purified by recrystallization from *n*-hexane/ethyl acetate 9:1: **3a**, m.p. 78–79 °C, yield 53%; **3b**, m.p. 88–89 °C, yield 52%; **3c**, m.p. 88–91 °C, yield 56%; **3d**, m.p. 86–91 °C, yield 55%.

Much lower yields were obtained under shorter reaction times; as an example, the data related to compound **3c** are reported: yields % 16h/6h = 55/8.

Compounds **4a–d** were obtained, as pure salts, by mixing the solution of the opportune dicarboxylic acid (10 mmol) with a solution of NaOH (20 mmol) in absolute ethanol, at room temperature under stirring. The products were dried at room temperature after filtration on a Buchner filter. 

Yields were about 97%–98%. The total yields of the synthetic process were as follows: **4a** = 40%, **4b** = 42%, **4c** = 45%, **4d** = 44%. 

### 3.3. ^1^H-NMR and ^1^H-^1^H COSY

The characterization of compounds **4a–d** was performed through ^1^H nuclear magnetic resonance spectroscopy (^1^H-NMR). A 90° pulse of 7.05 µs for the ^1^H nucleus together with a delay time of 2 s and 8 scans were employed. The same pulse duration for the ^1^H nucleus was employed during the COSY acquisition together with gradient pulses for selection, a delay time of 2 s, 4 scans and 256 experiments. The ^1^H-NMR spectra of all compounds in deuterium dioxide are reported in Figure 2.

### 3.4. Critical Micelle Concentration (CMC)

The CMCs of all synthesized surfactants were evaluated by investigating the conductivity behavior of the aqueous solution of each surfactant as a function of concentration. The solutions were prepared by dissolving the appropriate amounts of the surfactant in deionized water (Ω= 18.1 MΩ). The conductivity values are displayed as a function of molar concentration in Figure 4. 

The CMC values were evaluated from the intersection of the two linear regions observed in the experimental conductivity trends and are reported in Table 2. 

### 3.5. Diffusion Coefficient

The diffusion coefficient was determined at various concentrations for compounds **4a**–**d**. 

The pulsed field gradient nuclear magnetic resonance technique was used to determine the self-diffusion coefficients of the surfactants at 25.0 ± 0*.*5 °C by monitoring the ^1^H signal of the surfactants methylenic chain with the NMR spectrometer, equipped with a field gradient probe unit. A bipolar pulsed longitudinal eddy current delay (BPP-LED) with a stimulated echo pulse sequence was employed [21,22] in all the experiments. The length of the gradient pulse was kept constant and the gradient strength was varied in 16 steps from 0.7 to 32 G·cm^−1^ with a step length of 2.1 G·cm^−1^.

## 4. Conclusions

The disodium 2,3-dialkyl-1,2,3,4-butanetetracarboxylates compounds, with 8, 10, 12 and 14 carbon atoms in the alkyl chains, were synthesized by an eco-friendly procedure activated by microwave irradiation, which allowed us to obtain products of the desired architecture with high yield and purity as inferred by a ^1^H-NMR investigation. All compounds displayed surfactant properties; therefore, their critical micelle concentrations were determined by conductivity measurements. The observed CMC trend with the number of carbon atoms in the alkyl chains has been rationalized on the basis of literature findings. The translational diffusion coefficient of the micelles was determined, by means of self-diffusion-NMR, as a function of concentration. The model, used to fit the translational diffusion coefficient, describes the associative process as the mass-action model for compound **4a** and the pseudo-phase transition model for compounds **4b**–**d**. The micelle hydrodynamic radii, computed by means of the Stokes-Einstein equation, increase with the surfactant concentration for all compounds. 

The properties of these four gemini surfactants, in particular, the newly synthesized compounds **4a**, **4b** and **4d**, were investigated with the aim of acquiring a full understanding of their behavior in aqueous medium. The collected experimental data and, specifically, the very low values of critical micelle concentrations, might be of interest for potential applications in many fields of material science and technology, especially in the cosmetic field, where efficiency and safety are strictly connected.
